# First-in-class oral small molecule inhibitor of the tyrosine kinase ROR1 (KAN0439834) induced significant apoptosis of chronic lymphocytic leukemia cells

**DOI:** 10.1038/s41375-018-0113-1

**Published:** 2018-03-27

**Authors:** M. Hojjat-Farsangi, A. H. Daneshmanesh, A. S. Khan, J. Shetye, F. Mozaffari, P. Kharaziha, L.-S. Rathje, P. Kokhaei, L. Hansson, J. Vågberg, S. Byström, E. Olsson, C. Löfberg, C. Norström, J. Schultz, M. Norin, T. Olin, A. Österborg, H. Mellstedt, A. Moshfegh

**Affiliations:** 1 Department of Oncology-Pathology, Immune and Gene Therapy Lab, CancerCenterKarolinska (CCK), Karolinska University Hospital Solna and Karolinska Institute, Stockholm, Sweden; 2 Kancera AB, Solna, Sweden; 3 Cancer Research Center and Department of Immunology, Semnan University of Medical Sciences, Semnan, Iran; 4 Department of Hematology, Karolinska University Hospital Solna, Stockholm, Sweden

Development of new drugs with other mechanisms of action (MOA) in chronic lymphocytic leukemia (CLL) than those in clinical practice are highly warranted in spite of the recent clinical progress, especially compounds targeting tumor-specific molecules. Therapeutics interfering with signaling pathways controlling growth and survival of leukemic cells and bypass resistance to cytotoxic drugs is of special interest [1] and tyrosine kinase inhibitors (TKIs) in particular.

The receptor tyrosine kinase (RTK) ROR1 is normally expressed during embryogenesis but repressed in most adult tissues. We and others have shown high expression of ROR1 in CLL cells [2] including constitutive phosphorylation at tyrosine residues within the activation site of the TK domain [3]. ROR1 is of importance for e.g., tumor cell proliferation, survival, and metastasis [4]. Interfering with the ROR1 signaling pathway might be a successful approach to induce tumor cell apoptosis [2]. Inhibition of ROR1 by RNA interference (siRNA) and monoclonal antibodies (mAb) induced apoptosis of CLL cells [5, 6]. The PI3K/AKT/mTOR pathway seems to play an important role in ROR1 signaling [7]. Small molecule inhibitors targeting ROR1 has until now not been reported whereas humanized mAb, chimeric antigen receptor (CAR)-T cells, and BiTE targeting the extracellular part of ROR1 are in development [8, 9].

A small molecule, KAN0439834 (535 Da), targeting the TK domain of ROR1 was developed from a library of 110.000 compounds using fresh CLL cells (disease related cell phonotype selection procedure) specifically inhibiting phosphorylation of the TK domain (Supplementary Methods, Information and Figures).

Leukemic cells (PBMC 94–99% ROR1^+^ cells) from patients with non-progressive or progressive CLL (as defined by IWCLL criteria), as well as of patients with fludarabine resistant disease with or without del(17p) were analyzed for KAN0439834 induced cytotoxicity. A dose-dependent cytotoxicity was observed, which also included fludarabine resistant CLL cells with and without del(17p) (EC_50_ 250 nM) (Fig. 1a, b). There was no statistically significant difference comparing cells from patients with non-progressive and progressive disease. Selectivity for CLL cells compared to normal PBMC (ROR1^+^ cells <0.75%) was >60 folds at EC_50_. Apoptosis was confirmed by Annexin V/PI staining (Supplementary Figure 1A). A statistically significant correlation was noted between cytotoxicity and apoptosis (*r* = 0.80; *p* < 0.0001). KAN0439834 induced significant apoptosis of CLL B cells but not of normal T cells (Supplementary Figure 1B).

**Fig. 1:**
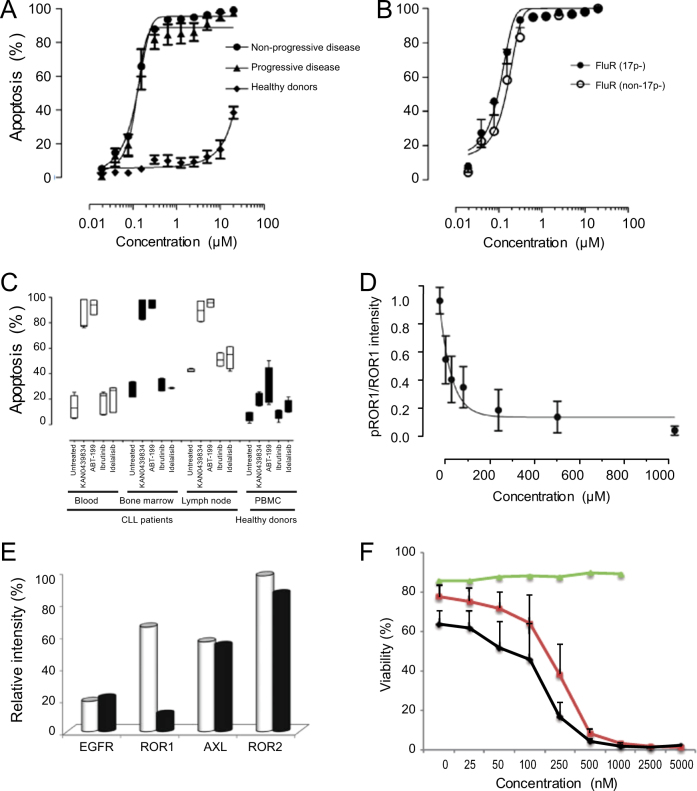
KAN0439834 induced significant cell death of CLL cells. **a** Cytotoxicity (24 h) of PBMC (Cell Titer Blue) from patients with non-progressive (*n* = 48) and progressive disease (*n* = 48) (IWCLL criteria), as well as from healthy controls (*n* = 23) (EC_50_ for CLL cells = 250 nM); **b** PBMC from patients with fludarabine refractory (FluR) disease with (*n* = 8) or without (*n* = 8) (del)17p ; **c** Apoptosis (Annexin V/PI) in CLL cells from blood (*n* = 9), bone marrow (*n* = 8), lymph nodes (*n* = 9) incubated (6 h) with KAN0439834 (250 nM), venetoclax (ABT-199) (50 nM), ibrutinib (250 nM), and idelalisib (100 nM), as well as PBMC from healthy donors (*n* = 6) (box diagrams); **d** ROR1 dephosphorylation (pROR1 relative to total ROR1) in CLL cells by KAN0439834 (30 min) (intensity values) (mean ± SEM) (*n* = 5); **e** Dephosphorylation of ROR1, ROR2, AXL, EGFR (Human phospho-RTK array) in untreated (□) and treated (■) CLL cell cultures incubated with KAN0439834 (250 nM) (pooled lysates from five patients) (intensity values of pROR1, pROR2, pAXL, and pEGFR spots); **f** Apoptosis (Annexin V/PI) (%) (mean ± SEM) in CLL cells (*n* = 3) after co-culture with HS-5 stromal cells (ROR1^−^) (24 h) in the presence of KAN0439834. Annexin V/PI was analyzed in CD45^+^/ROR1^+^ CLL cells and ROR1^−^ HS-5 cells. HS-5 cells alone (green line), CLL cells co-cultered with HS-5 cells (red line), and CLL cells alone (black line)

Next, we compared apoptosis induced by KAN0439834 with that of ibrutinib, idelalisib, and venetoclax (ABT-199) on CLL cells from different compartments (bone marrow, blood, lymph nodes) of the same patients. KAN0439834 and venetoclax induced a similar high degree of apoptosis of CLL cells from the three compartments while ibrutinib and idelalisib only killed CLL cells obtained from lymph nodes, but to a lower degree than KAN0439834 and venetoclax (Fig. 1c). KAN0439834 had a low killing capacity of healthy donor PBMC while venetoclax induced a higher degree of cytotoxicity of normal PBMC. Normal B and T cells were killed by venetoclax but not by KAN0439834 (Supplementary Figure 1C). The BCL-2, BCL-xL, MCL-1, and BAX proteins were downregulated in CLL cells following exposure to KAN0439834, PARP, as well as caspases 1 and 3 were cleaved confirming apoptosis (Fig. 2a). Six hours of drug exposure seemed to be sufficient to induce irreversible apoptosis of CLL cells (Supplementary Figure 2).

**Fig. 2:**
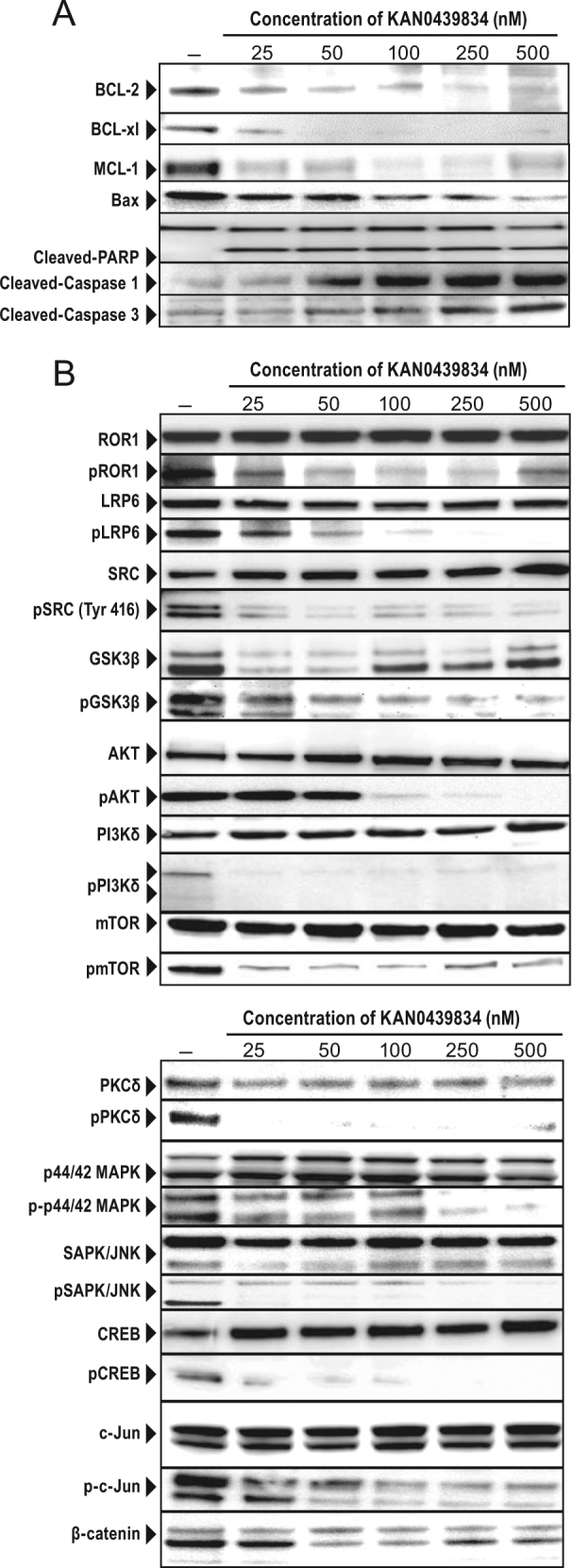
Pro-apoptotic proteins and signaling molecules in KAN0439834 treated cells. **a** CLL cells incubated with KAN0439834 (25–500 nM) (24 h) showed downregulation of BCL-2, Bcl-xl, MCL-1, and BAX, as well as cleaved PARP and caspase 1 and 3 [(one representative experiment out of three (WB)]; **b** CLL cells incubated with KAN0439834 (25–500 nM) (2 h) and tested for total and phosphorylated ROR1, LRP6, SRC, GSK-3β, AKT, PI3Kδ, mTOR, MAPK, JNK, CREB, c-JUN, β-catenin (one representative out of three-experiments)

KAN0439834 induced a dose-dependent dephosphorylation of ROR1 in CLL cells (WB) (Fig. 1d), which was confirmed using a human-phospho-RTK array. The assay detected the phosphorylated RTKs, ROR1, ROR2, AXL, and EGFR, which are expressed in CLL cells. KAN0439834 significantly reduced phosphorylation of ROR1 but not of ROR2, AXL, and EGFR (Fig. 1e).

Wnt5a (a ligand for ROR1) increased phosphorylation of ROR1 in a dose-dependent manner, but not SRC which might be activated by other kinases [10]. KAN0439834 dephosphorylated both ROR1 and SRC and inhibited Wnt5a-induced phosphorylation of ROR1 (Supplementary Figures 3A and B). Stromal cells are known to support the survival of CLL cells in the bone marrow and lymph nodes contributing to a more resistant cell phenotype as compared to CLL cells in the blood [11]. When CLL cells and HS-5 cells (ROR1^−^ stromal cells) were co-cultured, HS-5 cells could partially prevent apoptosis of CLL cells at low concentrations of KAN0439834, while at higher concentrations the presence of stromal cells had no effect (Fig. 1f). Moreover KAN0439834 suppressed Wnt5a and stromal cells induced ROR1 phosphorylation, which might then also contribute to inhibition of survival of CLL cells in addition to the direct apoptotic effect of KAN0439834 on the leukemic cells. These findings are in line with a report showing that the anti-ROR1 mAb cirmtuzumab inhibited Wnt5a-induced survival signals in CLL cells [12].

ROR1 and the co-receptor LRP6 may heterodimerize in CLL cells as part of signal transduction [13]. KAN0439834 dissociated the ROR1/LRP6 complex (Supplementary Figures 4A and B) and the complex proteins were dephosphorylated (Fig. 2b). SRC which binds to phosphorylated ROR1 [14] were also dephosphorylated and might be a starting event for the inactivation of ROR1 downstream signaling [14]. Molecules of both the canonical Wnt [GSK3β and β-catenin (decreased expression in the cytoplasm)] and non-canonical Wnt (PI3Kδ/AKT/mTOR) pathways were inactivated. PKCδ, MAPK, and SAPK/JNK pathways molecules were also dephosphorylated, as well as the transcription factors CREB and c-Jun (Fig. 2b). The initial activation event of ROR1 may differ between malignancies, but activation of the PI3K/AKT/mTOR pathway seems to be important irrespective of the initiation event [2].

KAN0439834 bound also to a few other kinases (KINOMEscan) but at high concentrations (10 µM) (Supplementary Methods and Information Table 3). However, no functional effects on normal PBMC (T and B cells) was noted and only minor side effects in mice were seen (see below).

The cytotoxic effects of KAN0439834 were also compared to 16 other kinase inhibitors in clinical or preclinical use (Supplementary Figure 5). EC_50_ for those inhibitors were >10 µM using the same target (CLL cells). Importantly, dephosphorylation of ROR1 was not observed by inhibitors such as bemcitinib (R428) (AXL inhibitor), idelalisib or ibrutinib, all of which, however, (as expected) dephosphorylated their respective targets (Supplementary Figures 6A and B).

Pharmacokinetics of KAN0439834 was analyzed in CD1 mice after a single oral dose (50 mg/kg) of KAN0439834 (Supplementary Figure 7). Six hours after the single dose, a plasma concentration of 800 nM was obtained, which should be sufficient to induce an irreversible apoptosis in CLL cells (see above). In NOD-SCID mice xenotransplanted with human CLL cells without del(17p) (no p53 mutations) there was a statistically significant reduction of CD45^+^/CD19^+^/ROR1^+^ cells in the spleen (flow cytometry) at a high dose of KAN0439834 but less pronounced at a low dose. A similar significant reduction of CD45^+^/CD19^+^/ROR1^+^ cells was also noted when CLL cells harboring del(17p) were transplanted but not as marked as for non-del(17p) CLL cells. A significant dose-dependent reduction of ROR1 expression was noted in both experiments, as well as reduction of phosphorylated ROR1. IHC analyses supported the results of flow cytometry (Supplementary Figure 8). Blood chemistry analyses showed no abnormal values except a slight increase in alanine transferase (ALT). Histopathology of organs showed small foci of necrotic areas in the liver and slight regenerative changes in the kidney tubular epithelium.

In summary, we report here on the development of a new class of cancer drug targeting ROR1 (KAN0439834)—a tyrosine kinase inhibitor. Novel drugs with other MOA are warranted to further improve the prognosis in CLL and related disorders. KAN0439834 may be a drug candidate also for other ROR1 expressing hematological and non-hematological tumors alone or in combination with other therapies.

## Electronic supplementary material

Supplementary Methods, Information and Figures
