# Supplementary material for: First-in-class oral small molecule inhibitor of the tyrosine kinase ROR1 (KAN0439834) induced significant apoptosis of chronic lymphocytic leukemia cells
Source: Leukemia. 2018 Mar 27;32(10):2291–5. doi: 10.1038/s41375-018-0113-1 (PMC6170396; doi:10.1038/s41375-018-0113-1)
Supplement: Supplementary file 1 — Supplementary Methods, Information and Figures [file 41375_2018_113_MOESM1_ESM.docx]

**Supplementary Methods, Information and Figures**

**Material and Methods**

**Patients and Healthy controls**

Leukemic cells were obtained from peripheral blood and for some experiments for bone marrow and lymph nodes from CLL patients. The diagnosis of CLL and criteria for progressive disease was established according to the IWCLL criteria. Characteristics of patients with fludarabine resistant disease are shown in **Table 1.** In **Table 2**, clinical characteristics of those patients are depicted where samples were obtained simultaneously from blood, bone marrow and lymph nodes. Peripheral blood mononuclear cells (PBMC) from age-matched healthy blood donors served as controls. Approval by the regional ethics committee (www.epn.se) was obtained as well as an oral and written informed consent from the donors in accordance with the Helsinki declaration.

**Isolation of mononuclear cells**

PBMC were isolated by Ficoll-Paque (GE Healthcare, Uppsala, Sweden) density gradient centrifugation ^1^ and used fresh or cryopreserved stored at -140°C in liquid nitrogen with fetal bovine serum and 10% dimethyl sulfoxide (DMSO) (Sigma-Aldrich Co, St Louis, MO, USA).

**Cell surface markers (flow cytometry)**

CLL cells and PBMC of healthy donors were stained as previously described ^2^. Briefly, 10^6^ PBMC were washed and suspended in 100 ul of PBS. Allophycocyanin (APC) conjugated anti-ROR1 (Miltenyi Biotec, Bergisch Gladbach, Germany), Alexa fluor 488-conjugated anti-CD5, PE/Cy7 conjugated anti-CD19, Alexa 700 conjugated anti-CD45 and PreCp conjugated anti-CD3 (BioLegend, San Diego, CA, USA) were added and incubated for 20 min at room temperature (RT). The cells were then washed with fluorescence-activated cell sorting (FACS) buffer and counted in a FACS Canto II flow cytometry (BD Biosciences, San Jose, CA, USA). The FlowJo software program (Tree Star Inc., Ashland, OR, USA) was used for analysis of cells.

**Purification of B and T cells**

B and T cells from PBMC of CLL patients were purified by passing through a nylon wool column (Biotest, Breiech, Germany) and enriched by immunomagnetic beads using Midi-MACS columns with anti-CD19 and anti-CD3 conjugated MACS Microbeads (Miltenyi Biotec) as previously described ^3^. Purity of cells was checked by flowcytometry (94-99%).

**SDS-PAGE and Western blot**

Western blot experiments were performed as previously described ^1^. CLL cells or healthy PBMC were lysed on ice for 30 min in buffer containing 1% Triton X-100, 150 mM NaCl, 50 mM Tris-HCl, 5 mM EDTA, 1% protease inhibitor cocktail (Sigma-Aldrich) and phosphatase inhibitors (Roche Ltd, Basel, Switzerland) and centrifuged at 13000 rpm. Supernatants were collected and protein concentration measured by the BCA Protein Assay Kit (ThermoFisher Scientific, IL, USA). Twenty μg of the lysate were loaded onto 10% Bis-Tris SDS-PAGE gel (ThermoFisher Scientific) and run at 160 V and 150 mA for 2 h. Electrophoresed proteins were transferred to PVDF membranes (Millipore Corporation, MA, USA) and blotted at 45 V and 145 mA for 1.5 h in Transblot cell (ThermoFisher Scientific) at RT. Membranes were blocked in blocking buffer (5% bovine serum albumin (BSA) (Santa Cruz Biotechnology, CA, USA) in TBS with 0.1% Tween 20 (TBS-T) at RT for 2 h. Membranes were probed with the respective primary antibodies overnight at 4°C and washed five times for 10 min in TBS-T at RT and incubated with secondary antibody conjugated with peroxidase (Dako Cytomation, Glostrup, Denmark) for 1.5 h in blocking buffer at RT. Membranes were washed five times and developed using the ECL chemiluminescence detection system (GE Heathcare, Uppsala, Sweden). The following antibodies were used; ROR1 (R&D Systems, Minneapolis, MN, USA), phospho (p) ROR1 (Tyr 641, 646 and Ser 652) (**Supplementary Methods Information I**), myeloid cell leukemia (MCL)-1, poly ADP ribose polymerase (PARP), B-cell lymphoma (BCL)-2, BCL-xL, caspase 3, Bax, LDL receptor related protein (LRP) 6, pLRP6 (Ser 1490), SRC, pSRC (Tyr 419), glycogen Synthase Kinase (GSK) 3β, pGSK3β (Ser 9), β-catenin, AKT, pAKT (Ser 473), PI3K p110δ, mTOR, p-mTOR (Ser 2448), protein Kinase C (PKC) δ, pPKCδ (Tyr 311), p44/42 mitogen-activated protein kinase (MAPK) (Erk1/2), p44/42 MAPK (Erk1/2) (Thr 202/Tyr 204), stress-activated protein kinase/c-Jun N-terminal kinase (SAPK/JNK), pSAPK/JNK (Thr 183/Tyr 185), cAMP response element-binding protein (CREB), pCREB (Ser 133), c-Jun, p-c-Jun (Ser 73) (Cell Signaling Technology, MA, USA), and p-PI3k p110δ (Tyr 485) (Santa Cruz). For loading control, membranes were probed for β-actin (Sigma-Aldrich). Densitometric quantification was carried out using the Image J1.44p software (National Institute of Health, USA). Ratios were calculated between phosphorylated and total protein.

**ROR1 tyrosine kinase inhibitor (KAN0439834)**

A recombinant protein consisting of the TK domain of ROR1 was subjected to a high-throughput screening (HTS) campaign of 110 000 diverse compounds using an assay detecting compounds that prevented phosphorylation of a substrate peptide, see below. 614 hits from the primary screen were tested for inhibition of ROR1 activity in 11 concentrations ranging from 3.4 nM to 200 µM in the HTRF® KinEASE assay using the same protocol as described above. The hits were clustered by hierarchical clustering based on Tanimoto distances calculated from structural fingerprints and the clustering was refined using manual curation and sub-structure searching to define hit series and singletons. Each cluster and singleton was thoroughly reviewed and scored based on parameters such as drug-likeness, IC_50_, dose-response curve shape, analytical data, synthetic amenability, and ligand efficiency. Based on the evaluation, six clusters and four singletons were prioritized for further analysis and 160 analogues to the compounds in the prioritized clusters and singletons, were identified from our compound library consisting of around 270,000 compounds and tested in the ROR1 kinase assay. All compounds showing a dose-response were subjected to analytical QC assays (identity, purity, chemical stability and kinetic solubility using reversed phase HPLC with UV and ESI-MS detection).

The further development was focused on the singleton KAN0173631 which combined characteristics suitable for a chemistry development program with an ability to induce apoptosis of primary CLL cells and selectivity towards PBMC from healthy donors. The further development mainly relied on phenotypic screening (cell viability assay, see below) using fresh CLL cells from patients. More than 2000 analogues to KAN0173631 were synthesized and tested for activity in the cell viability assays (PBMC from CLL patients and healthy volunteers) and selected compounds were further characterized for effects on ROR1 phosphorylation, apoptosis (Annexin V/PI) as well as for downstream apoptotic signaling molecules. The development resulted in the compound KAN0439834 (MW=535) which shows a high degree of “druglikeness” as it contains no apparent toxicophores and adheres to criteria typical for an orally active drug, the so-called Lipinski’s rule-of-Five ^4^. The calculated cLogD_7.4_ is 2.0. The drug is orally bioavailable (F=35% in the mouse) and showed intrinsic clearance values in hepatocytes of CLint=14 µl/min/10^6^ cells (human) and CLint=20 µl/min/10^6^ cells (mouse) respectively. Plasma stability T_1/2_ was >2 h in both human and mouse plasma. **Supplementary Methods and Information II**.

**Biochemical ROR1 kinase activity assay**

The assay used in the HTS campaign was a homogenous-time resolved fluorescence HTRF® KinEASE tyrosine kinase assay kit (Cisbio, Cedex, France) in 384-well micro plates (Corning, NY, USA) where the ROR1 activity was measured using a universal biotinylated TK-peptide substrate in combination with an anti-phosphotyrosine specific monoclonal antibody labeled with Eu^3+^-cryptate and detected by XL665 labeled streptavidin (Cisbio, Cedex, France). The final concentrations of the reaction mix were 100 µM ATP, 285 nM TK substrate-biotin, 70 ng protein/well of ROR1 in 15 mM Tris-HCl pH 7.5, 0.01% Tween 20, 10 mM MgCl2, 75 nM SEB (a proprietary substrate stabilizing buffer; Cisbio, Cedex, France), and 2 mM DTT. After 3.5 h incubation at room temperature, 10 µL of stop solution (36 nM XL665 and europium-labeled anti-PiTK antibody according to the manufacturer) was added. The plate was incubated over-night in dark at 4°C and fluorescence was read at 615 and 665 nm following excitation at 340 nm using a VictorV2 plate reader (Perkin Elmer, CA, USA).

**Cellular cytotoxicity assay**

The CellTiter-Blue® cell viability assay (Promega, Madison, WI, USA) was used to measure cytotoxic effects of drugs according to the manufacturer´s instruction. Stock solutions (10 mM in DMSO) of compounds were serially diluted 1:2 in 11 concentrations and subsequently 50 nl/well were dispensed into assay plates (384-well black/clear, Sigma-Aldrich) using an acoustic dispenser (EDC Biosystems, Fremont, CA, USA). Final starting concentration in the assay was 20 µM (0.2% DMSO) for test compounds. 50,000 PBMC/well from CLL patients or healthy control donors were seeded in the assay plates and incubated for 24 h at 37^○^C. After incubation, CellTiter Blue reagent was added (5 µl/well) and plates were incubated for 2 h. Plates were read in an Envision fluorescence reader (PerkinElmer) with Ex544 nm/Em590 nm. Results were calculated as % cytotoxicity compared to background (cells treated with 0.2% DMSO).

**Apoptosis assay (flow cytometry)**

One ml (2 x 10^6^ cells/ml) of PBMC from CLL patients and controls were seeded in each well of 24-well plates with and without compounds for 24 h. Cells were collected, washed with PBS, suspended in 100 μl of Annexin-V binding buffer (BD Biosciences) containing FITC-conjugated Annexin-V and PI (BD Biosciences) and incubated at RT in the dark for 20 min. After 20 min of incubation, 150 μl fresh Annexin-V binding buffer was added. Cells were then stained with alexa fluor-700 conjugated anti-CD45, PE/Cy7 conjugated anti-CD19 and PreCp conjugated anti-CD3 antibodies (BioLegend). Viable cells were identified as the double negative Annexin-V and PI population. Samples were analyzed by a FACS Canto II Flow-cytometer (BD Biosciences) ^5^.

**Gene microarray analysis**

Total RNA was purified from the untreated and treated (250 nM of KAN0439834 for 6 h) MEC-II CLL cell line (DSMZ, Braunschweig, Germany) according to the Qiagen protocol (WVR, Stockholm, Sweden). RNA purity and concentration were measured by Bioanlyzer (Agilent, Santa Clara, CA, USA). After extraction, mRNA was subjected to analysis for global gene expression using Affymetrix GeneChip® Human HT U133 Array (Affymetrix, Santa Clara, CA, USA). Data were analyzed by the Genespring software (Agilent, Santa Clara, USA). Supervised clustering was performed on geometric means of the respective arrays by using complete linkage hierarchical clustering with the Euclidean distance between each sample arrays as the distance metric ^6^.

**Apoptosis of CLL cells co-cultured with stromal cells**

The human stromal cell line HS-5 (ROR1 negative) and fresh CLL cells were treated with KAN0439834 (25-500 nM) in RPMI-1640 containing 10% FBS, seeded into 24-wells plate at a ratio of 1:5 and incubated at 37^○^C in 5% CO_2_ for 24 h. Cells were harvested and stained for Annexin V/PI in ROR1^+^/CD45^+^/CD19^+^ gated cells. Untreated and treated HS-5 and CLL cells alone respectively served as controls.

**Apoptosis in CLL cells treated with Wnt5a**

CLL cells were cultured in RPMI 1640 supplemented with 10% FBS and treated with 50 and 250 nM of KAN0439834 for 2 h. 25 or 50 ng/ml of recombinant Wnt5a (OriGene Technologies Inc. Rockville, MD, USA) were added and incubated for 30 min before preparation of lysates and for 24 h before staining with Annexin V/PI.

**Phosphorylation of RTKs in CLL cells**

Phosphorylated RTKs in CLL cells were analysed by the Proteome Profiler Array Kit (Human-phospho-RTK array) (R&D system) according to the manufacturer’s protocol. Briefly, membranes were blocked and incubated with CLL cell lysates (700 μg) overnight at 4^°^C, washed 3 times with buffer and incubated with the secondary antibody (HRP-conjugated anti-phosphotyrosine) for 2 h at RT. Membranes were washed 3 times with buffer before development with the ECL western blotting detection reagent (GE Healthcare). RTK spots were visualized using the ECL chemiluminescence detection system (GE Heathcare). The degree of phosphorylated RTKs was quantified by densitometry using ImageJ software (National Institutes of Health, Bethesda, MD, USA) and normalized to the internal phosphotyrosine-positive control spots.

**Off-target binding of KAN0439834**

Off-target binding of KAN0439834 (selectivity) was measured by KINOMEscan™ (DiscoverX, San Diego, CA, USA) profiling as previously described ^7^. In this profiling, competition assays are used to measure the binding interactions between a test compound (KAN0439834) and 456 human kinases and disease relevant mutant variants (395 non-mutant kinases). ROR1 or ROR2 were not among the 456 kinases. The competition assay measures the ability of test compounds to prevent binding of the kinases to active-site directed immobilized ligands. The binding interactions are presented as percent remaining control ligand (% C). Lower numbers indicate stronger binding. A selectivity score can be calculated as a ratio between the number of non-mutant kinases and % C values below a selected cutoff (e.g. 1%) divided by the total number of non-mutant kinases tested.

**Immunofluorescent (IF) assay**

The immunofluorescent (IF) assay was performed as previously described ^5^. Briefly, CLL cells were seeded on slides and fixed with 4% paraformaldehyde for 10 min, washed with PBS (pH 7.4), and blocked for 1 h in blocking buffer (2% bovine serum albumin (BSA), 1% Tween 20 and 0.01% sodium azide in PBS). The slides were incubated with 1 ug/ml of antibodies against ROR1 and LRP6 (Sigma-Aldrich), ROR2 (Abcam, Cambridge, MA, UK) as well as an unspecific mAb (mouse and rabbit IgG) (eBioscience) overnight at 4^°^C. Slides were then washed three times with PBS and incubated with Alexa Flour 488-conjugated goat anti-mouse and rabbit IgG (1:500) (ThermoFisher Scientific, Uppsala, Sweden) for 1 h. After three washes in PBS, the nuclei were stained using VectaShield H-1000 mounting media containing DAPI (Vector Laboratories, Burlingame, CA). A Zeiss Axioplan2 fluorescence microscope (Oberkochen, Germany) equipped with a 63X objective lens and ZEN software (Carl Zeiss Microscopy, Germany) was used to document the cell staining.

**Proximity ligation assay (PLA)**

PLA test is applied to analyse approximation of proteins in a cell or on the cell surface. Untreated and KAN0439834 treated (6 h) CLL cells were seeded onto slides and fixed with 4% formaldehyde, washed 3 times with PBS and blocked with blocking buffer (2% BSA, 0.1& Tween 20 and 0.01% azide) for 1 h at RT. Cells were then incubated overnight at 4°C with ROR1 and LRP6 primary antibodies (1:100) (Sigma-Aldrich). PLA was performed using the Duolink^®^ In Situ Red Starter Kit Mouse/Rabbit (Sigma-Aldrich) according to the manufacturer’s protocol. After overnight incubation with the primary antibodies, slides were washed and PLA probes added and incubated at 37°C for 1 h. Ligation reaction was done for 30 min after washing and the amplification process was run for 100 min. Cells were washed and nuclei counterstained with VectaShield H-1000 mounting media containing DAPI (Vector Laboratories, Burlingame, CA, USA).

**Pharmacokinetic (PK) studies in mice**

Blood was collected from male CD1 mice at different time points after a single oral dose of KAN0439834 in suspension at 50 mg/kg. Sampling was performed by semi serial bleeding with 3 samples/time point (in total 9 mice). Plasma was prepared by centrifugation and samples were frozen until further analysed by LC-MS/MS. (All PK studies were done at ChemPartner, Shanghai, China).

**Xenograft studies in mice**

All animal experiments were approved by the local animal ethical committee of Stockholm, Sweden. Animal care was in accordance with institutional guidelines. Two different animal studies were performed: study I and II.

In study I, PBMC from a progressive CLL patient without 17p deletion was used. 500 x 10^6^ cells/ml for intravenous (iv) administration and 125 x 10^6^ cells/ml for intraperitoneal (ip) injection, were suspended in PBS and injected to 8-weeks old NOD-SCID mice (Charles River, Lyon, France), in a volume of 0.2 ml for iv and 0.4 ml for ip administration. Oral treatment twice daily with KAN0439834 or matched vehicle started 7 days after cell injection. KAN0439834 was diluted in 7% DMSO, 5% ethanol, 25% HPβCD in PBS. The second dose was administered 8 hours after the first. For the low dose, eight mice received 10 mg/kg twice daily and for the high dose 8 mice received 25 mg/kg as the morning dose followed by 12.5 mg/ml of KAN0439834 in the evening, for 7 days. Four control mice received the vehicle only for 7 days.

In study II, PBMC from a progressive CLL patient with 40% of leukemic cells exhibiting 17p deletion were suspended in PBS at a concentration of 500 x 10^6^ cells/ml for iv and 125 x 10^6^ cells/ml for ip administration to 8-weeks old NOD-SCID mice (Charles River, Lyon, France). Treatment with KAN0439834 in 0.5% Tween 80 and 1% glycerol started 7 days after the cell injection and continued for 14 days. Eight mice were dosed orally with 60 mg/kg once daily, during two weeks (low dose). Another 8 mice received 40 mg/kg twice daily of KAN0439834 during the first week followed by 60 mg/kg daily during the second week (high dose). Eight control mice received the vehicle only.

Mice were sacrificed on day 14 (study I) and day 21 (study II) after cell injection, i.e. after 7 and 14 days of treatment, respectively. During the whole experiment, body weight was measured daily. Spleens were harvested and used for IHC and flow cytometry. Single cell suspension from spleens was prepared using single cell strainer (BD Biosciences), collected in PBS containing 1% BSA, centrifuged, washed in the same buffer and analysed by flow cytometry and Western blot.

**Immunohistochemistry (IHC) staining**

Immunohistochemistry staining of formalin-fixed, paraffin-embedded (FFPE) mice spleens was carried out as previously described ^8^. Briefly, FFPE spleen tissues were cut in 2 µm sections, mounted onto adhesive-coated glass slides and human CD45^+^ expressing cells were analysed. Before CD45 staining, slides were stained with hematoxylin-eosin. After de-paraffinization and re-hydration, slides were heated in a microwave oven in citrate buffer and incubated with 10% hydrogen peroxide for 30 min and 1% bovine serum albumin in tris-buffered saline for 30 min to block endogenous peroxidase and prevent non-specific binding. The slides were then incubated with a mouse anti-human CD45 mAb (BD Biosciences) (1:200) in a moist chamber at 4^°^C overnight and with an anti-mouse secondary antibody (1:200) for 1 h. Finally, the slides were counterstained with Mayer’s hematoxylin and dehydrated before mounting. Normal mice spleens were used as control.

**Statistics**

EC_50_ values were obtained by non-linear curve fitting to Hills equation using GraphPad Prism of the Accord HTS software (Accelrys Inc., San Diego, CA, USA), ANOVA and T-test. GraphPad Prism version 5 (GraphPad Software, Inc., La Jolla, CA, USA) was used for statistical analysis of flow cytometry data. Statistical calculation of normalized intensity values for each protein was done by one-way ANOVA test followed by post-hoc Tukey’s honest significant difference test (HSD). The cut-off for significance was set to p < 0.05.

**References**

1. Hojjat-Farsangi M, Khan AS, Daneshmanesh AH, Moshfegh A, Sandin A, Mansouri L*, et al.* The tyrosine kinase receptor ROR1 is constitutively phosphorylated in chronic lymphocytic leukemia (CLL) cells. *PLoS One* 2013; **8**(10)**:** e78339.

2. Daneshmanesh AH, Hojjat-Farsangi M, Khan AS, Jeddi-Tehrani M, Akhondi MM, Bayat AA*, et al.* Monoclonal antibodies against ROR1 induce apoptosis of chronic lymphocytic leukemia (CLL) cells. *Leukemia* 2012 Jun; **26**(6)**:** 1348-1355.

3. Kiaii S, Kokhaei P, Mozaffari F, Rossmann E, Pak F, Moshfegh A*, et al.* T cells from indolent CLL patients prevent apoptosis of leukemic B cells in vitro and have altered gene expression profile. *Cancer Immunol Immunother* 2013 Jan; **62**(1)**:** 51-63.

4. Lipinski CA, Lombardo F, Dominy BW, Feeney PJ. Experimental and computational approaches to estimate solubility and permeability in drug discovery and development settings. *Adv Drug Deliv Rev* 2001 Mar 1; **46**(1-3)**:** 3-26.

5. Hojjat-Farsangi M, Ghaemimanesh F, Daneshmanesh AH, Bayat AA, Mahmoudian J, Jeddi-Tehrani M*, et al.* Inhibition of the receptor tyrosine kinase ROR1 by anti-ROR1 monoclonal antibodies and siRNA induced apoptosis of melanoma cells. *PLoS One* 2013; **8**(4)**:** e61167.

6. Hogfeldt T, Bahnassy AA, Kwiecinska A, Osterborg A, Tamm KP, Porwit A*, et al.* Patients with activated B-cell like diffuse large B-cell lymphoma in high and low infectious disease areas have different inflammatory gene signatures. *Leuk Lymphoma* 2013 May; **54**(5)**:** 996-1003.

7. Liu F, Wang J, Yang X, Li B, Wu H, Qi S*, et al.* Discovery of a Highly Selective STK16 Kinase Inhibitor. *ACS Chem Biol* 2016 Jun 17; **11**(6)**:** 1537-1543.

8. Woopen H, Pietzner K, Richter R, Fotopoulou C, Joens T, Braicu EI*, et al.* Overexpression of the epithelial cell adhesion molecule is associated with a more favorable prognosis and response to platinum-based chemotherapy in ovarian cancer. *J Gynecol Oncol* 2014 Jul; **25**(3)**:** 221-228.

**Supplementary Methods and Information, Table 1.** Main characteristics of
fludarabine resistant patients with or without chromosome 17p deletion.

| **CLL code** | **Age** | **Gender** | **White blood cell count** | **FISH analysis*** |
| --- | --- | --- | --- | --- |
| 5390 | 57 | M | 39.7 | 17p- (36%) |
| 5397 | 58 | M | 30 | 17p- (59%) |
| 5398 | 72 | F | 35 | 17p- (39%) |
| 5419 | 76 | F | 17 | 13q- (59%) |
| 5196 | 81 | M | 146 | trisomy 12 (77%) |
| 5238 | 67 | M | 102 | 17p- (10%) |
| 5311 | 80 | F | 114 | 13q- (92%) |
| 5006 | 63 | M | 192 | 17p-** |
| 5090 | 74 | M | 51 | 17p- (71%) |
| 5169 | 79 | M | 270 | trisomy 12 (59%) |
| 5199 | 76 | M | 39 | trisomy 12 (86%) |
| 5203 | 55 | M | 53 | 17p- (77%) |
| 5207 | 75 | F | 125 | 17p- (35%) |
| 5317 | 61 | M | 50 | 13q- (68%) |
| 5312 | 75 | M | 60 | trisomy 12 (72%) |
| 5269 | 59 | F | 67 | 11q-** |

* FISH analyses were done within 1-2 months before sampling. TP53 sequencing was always added in patients without del (17p).

** Information on percentage missing.

**Supplementary Methods and Information, Table 2.** Clinical characteristics of CLL patients where samples were obtained simultaneously from the blood, bone marrow and lymph node and tested for KAN438934 induced cytotoxicity (Figure 1C).

| **Patient code** | **Age** | **Gender** | **Disease activity at testing** |
| --- | --- | --- | --- |
| 5167 | 75 | M | Non-progressive |
| 5274 | 73 | M | Non-progressive |
| 5350 | 75 | F | Progressive |
| 5533 | 64 | M | Non-progressive |
| 5539 | 82 | F | Progressive |
| 5507 | 66 | M | Progressive |
| 5541 | 76 | F | Progressive |
| 5546 | 58 | M | Non-progressive |
| 5414 | 65 | F | Progressive |

**Supplementary Methods and Information, Table 3.** Examples of off-target binding
at 10 μM KAN0439834 defined by KINOMEscan™ profiling (DiscoverX).

| **Pathway** | **Gene name** | **% of Ctrl at 10 μM** |
| --- | --- | --- |
| **PDGF signaling pathway** | MAPK15 | 0.45 |
|  | PDGFRβ | 0.05 |
|  | JAK3 | 0 |
|  |  |  |
| **Interleukin signaling pathway** | MKNK2 | 0.3 |
|  | MAPK15 | 0.45 |
|  | JAK3 | 0 |
|  |  |  |
| **Cyclin-dependent kinases** | CDK4 | 0.05 |
|  | CDK7 | 0.1 |
|  | CDKL2 | 0 |

KAN0439834 has been tested for binding to 456 different human kinases and disease relevant mutant variants at conc. of 10 μM. The binding interactions are presented as % remaining control ligand (% Ctrl). Lower numbers indicate stronger binding. In total 15 kinases showed < 0.1% of Ctrl at 10 μM KAN0439834. PDGF: platelet-derived growth factor, MAPK: mitogen-activated protein kinase, PDGFR β: platelet-derived growth factor receptor beta, JAK: Janus kinase, MKNK: MAP kinase-interacting serine/threonine-protein kinase, CDK: cyclin dependent kinase, CDKL: cyclin dependent kinase-like.

**Supplementary Methods and Information I**

**Production of anti-phospho-ROR1 polyclonal antibodies**

An anti-phospho-ROR1 (pROR1) polyclonal antibody was produced by Agrisera AB (Vannas, Sweden) by immunization of rabbits with a phospho-peptide derived from the tyrosine kinase domain of ROR1 phosphorylated at tyrosine residues 641 and 646 and serine residue 652 (EIY(p)SADYY(p)RVQSKS(p)LLPIR). After a standard immunization protocol, pROR1 antibodies were purified by affinity column chromatography using the phospho-peptide as ligand. Non-phospho-ROR1 antibodies were removed from the first affinity chromatography effluent using unphosphorylated peptide. Titration of pROR1 antibodies in the presence of phospho antigen and non-phospho antigen is shown in the Supplementary information I, Figure 1a. The specificity of the pROR1 antibodies was also tested using a recombinant auto-phosphorylated ROR1 protein (55 kDa) (kind gift from Dr Daniela Ungureanu, University of Tampere, Finland), CLL lysates expressing phosphorylated (ROR1) (130 kDa) and a protein phosphatase enzyme (New England BioLabs, Hitchin, Hertfordshire, UK). Briefly, the specificity of the anti-pROR1 polyclonal antibody was analysed before and after treatment of CLL cells and the recombinant phosphorylated intracellular ROR1 protein using a protein serine/threonine/tyrosine phosphatase as previously described ^1^. Briefly, 2 µg recombinant protein or 40 µg lysate from CLL cells without phosphatase inhibitors was incubated with 400 units of the phosphatase enzyme in the presence of protein metallo-phosphatases and MnCl_2_ at 37 °C for 30 min. Treated and untreated samples were analysed for ROR1 phosphorylation in Western blot (Supplementary information I, Figures 1b and c).

The reactivity of the pROR1 antibody was also tested using a peptide from the ROR1-KNG domain ^2^, a recombinant protein of the full length extracellular part and a full length ROR1 protein as well as a full length recombinant CEA protein (Protein Sciences Corp., Meriden, Mass., USA) ^3^ in ELISA (wells coated with 1 µg of each protein) (Supplementary information I Figure 1d).

**Supplementary Methods and Information I, Figure 1.** Specificity analysis of the anti-human phospho-ROR1 polyclonal antibody. (**A**) Titration of the anti-phospho-ROR1 antibody in ELISA. The pROR1 antibody detected a ROR1 phospho peptide but not a non-phospho-ROR1 peptide. (**B**) Western blots of a recombinant auto-phosphorylated intracellular ROR1 protein (kind gift of Dr Daniela Ungureanu, Tampere University, Finland) (55 kDa) (-) before and after (+) treatment with a protein phosphatase enzyme and (+) after treatment with the protein phosphatase enzyme. (**C**) CLL lysates from two patients (CLL 5628, CLL 5640) before (-) and after (+) treatment with a protein phosphatase enzyme for 30 min at 37°C. PVDF blots were stripped and reprobed with a rat polyclonal antibody against a full length ROR1 protein (OriGene Technologies, Inc., Herford, Germany) to confirm ROR1 reactivity. (**D**) Reactivity of the pROR1 polyclonal antibody in ELISA using a ROR1 peptide from the extracellular part (KNG) as well as recombinant extracellular and full length ROR1 protein. Recombinant CEA was used as a control.

In summary, the pROR1 polyclonal antibodies recognized the immunizing phospho-ROR1 peptide and not a non-phosphorylated peptide, a phosphorylated intracellular recombinant ROR1 protein including the TK domain of ROR1 and phosphorylated ROR1 protein derived from CLL cells but not after phosphatase treatment (dephosphorylation). Moreover, in ELISA the antibody only recognized the full length ROR1 protein but not the extracellular ROR1 protein and not a control protein.

**References**

1. Hojjat-Farsangi M, Khan AS, Daneshmanesh AH, Moshfegh A, Sandin A, Mansouri L*, et al.* The tyrosine kinase receptor ROR1 is constitutively phosphorylated in chronic lymphocytic leukemia (CLL) cells. *PLoS One* 2013; **8:** e78339.

2. Daneshmanesh AH, Hojjat-Farsangi M, Khan AS, Jeddi-Tehrani M, Akhondi MM, Bayat AA*, et al.* Monoclonal antibodies against ROR1 induce apoptosis of chronic lymphocytic leukemia (CLL) cells. *Leukemia* 2012 Jun; **26:** 1348-1355.

3. Staff C, Magnusson CG, Hojjat-Farsangi M, Mosolits S, Liljefors M, Frodin JE, et al. Induction of IgM, IgA and IgE antibodies in colorectal cancer patients vaccinated with a recombinant CEA protein. J Clin Immunol 2012 Aug; 32: 855-865.

**Supplementary Methods and Information II**

**Development of ROR1 tyrosine kinase inhibitors**

***High Throughput Screening***

A library of 110,000 different chemical compounds was screened at 10 µM concentrations to identify those with ROR1 inhibitory activity *in vitro* using a recombinant intracellular ROR1 protein (amino acids 428-937), (Carna Biosciences Inc, Kobe, Japan) applying a homogenous-time resolved fluorescence HTRF® KinEASE tyrosine kinase assay kit (Cisbio, Cedex, France) in 384-well micro plates (Corning, NY, USA). HTRF combines standard FRET (fluorescence resonance energy transfer) technology with time-resolved fluorescent measurement, which eliminates short-lived backgrounds. ROR1 activity was measured using a universal biotinylated TK-peptide substrate in combination with an anti-phosphotyrosine specific monoclonal antibody (Millipore Corporation, Bedford, MA, USA) labeled with Eu^3+^-cryptate and detected by XL665 labeled streptavidin (Cisbio, Cedex, France). The kinase assay was an end-point assay with four additions: 20 nl 5 mM DMSO compound stock; 4 µl recombinant ROR1 in enzyme buffer, 6 µl ATP and TK substrate-biotin mix, 10 µl XL665, and cryptate stop-detection mix. Staurosporine, a non-selective kinase inhibitor, and DMSO were used as positive and negative controls, respectively. Twenty nl from the 5 mM DMSO stock solution and compounds (10 µM final concentration) from the library were spotted into the 384-well assay plates using an EDC Biosystem acoustic dispenser prior to addition of the ROR1 mix which was followed by a 30 min pre-incubation at room temperature. The reaction was initiated by the addition of the ATP/TK substrate-biotin mix. The final concentrations of the reaction mix were 100 µM ATP, 285 nM TK substrate-biotin, 70 ng protein/well of ROR1 in 15 mM Tris-HCl pH 7.5, 0.01% Tween 20, 10 mM MgCl2, 75 nM SEB (a proprietary substrate stabilizing buffer; Cisbio, Cedex, France), and 2 mM DTT. After 3.5 h incubation at room temperature, 10 µL of stop solution (36 nM XL665 and europium-labeled anti-PiTK antibody according to the manufacturer) was added. The plate was then incubated over-night in dark at 4°C and fluorescence was then read at 615 and 665 nm following excitation at 340 nm using a VictorV2 plate reader (Perkin Elmer, CA, USA).

***Hit confirmation***

614 hits from the primary screen were tested for inhibition of ROR1 activity in 11 concentrations ranging from 3.4 nM to 200 µM in the HTRF® KinEASE assay using the same protocol as described above. Compounds (10 μM DMSO stocks) were diluted using a JANUS liquid handling station (Perkin Elmer) in a 3-fold serial dilution in DMSO in 384-well micro plates (Greiner, UV-star). An aliquot of 200 nl of each dilution was transferred to two assay plates (each concentration was assayed in duplicate) using an EDC Biosystems acoustic dispenser. 248 compounds showing a dose-response were subjected to analytical assays (identity, purity, chemical stability and kinetic solubility using reversed phase HPLC with UV and ESI-MS detection). In order to identify compounds active via a redox mechanism, the confirmed hits were assayed in dose-response in a 384-well redox-assay as described previously ^9^. A compound with verified redox activity and DMSO were used as positive and negative controls, respectively. The confirmed hits were clustered by hierarchical clustering based on Tanimoto distances calculated from structural fingerprints. The resulting clustering was refined using manual curation and sub-structure searching to define hit series and singletons. Each cluster and singleton was thoroughly reviewed and scored based on parameters such as drug-likeness, IC_50_, dose-response curve shape, analytical data, synthetic amenability, ligand efficiency, redox activity and whether the structure may be a pan-assay interference compound, a so-called PAIN ^10^. Based on the evaluation, six clusters and four singletons were prioritized for further analysis.

To distinguish between ATP competitive and non-competitive mechanisms of inhibition, 11 representative compounds from the prioritized clusters and singletons were tested in dose-response for ROR1 at ATP concentrations of 30 µM and 300 µM, i.e. 0.3-fold and 3-fold the ATP concentration used in HTS and hit confirmation assays. At ATP concentrations above 300 µM ROR1 started to be inhibited by ATP itself and was not used. An IC_50_ shift to higher IC_50_ values at the higher ATP concentration was used as an indication of an ATP competitive mechanism of action. All tested compounds appeared to be ATP competitive except one singleton (KAN0242909).

The same 11 representative compounds were also subjected to jump-dilution experiments ^11^ in order to identify irreversible inhibitors. Samples with compound concentrations 10 times higher than their corresponding IC_50_ and at 100 times higher enzyme concentration than in the standard assay were pre-incubated for 30 min. The samples were then rapidly diluted 100-fold with ATP/substrate mix to start the enzymatic reaction. Aliquots from the reaction mix were withdrawn at 8 different time points (in the range 0-240 min). The reaction was then stopped and the samples were read. The basic assay setup was the same as in the primary screen and hit confirmation experiments except that dilutions were different and enzyme concentration higher. All eleven compounds appeared to be fully reversible.

The same 11 representative compounds were further subjected to a Dynamic Light Scattering (DLS) study to test solubility of the compounds in DMSO at 10 mM and in the assay buffer at 1 and 10 µM. IC_50_ values in the ROR1 activity assay of the compounds were in the range of 1-10 µM. Compounds that self-associated and formed micelles in the assay buffer are likely to sequester the target enzyme and thus inhibit the enzyme non-specifically ^12^ were excluded. The DLS study was performed using DynaPro MS800 (Wyatt Corp., Santa Barbara, CA, USA) equipped with a 248-channel Multi-Tau USB correlator and a temperature control unit. The DMSO stock solutions were free from particles and no micelles were detected in the assay buffer solutions at 1 and 10 µM concentrations.

***Hit to lead development and lead optimization***

In total 160 compounds, analogues to the compounds in the prioritized clusters and singletons, were identified from our compound library consisting of around 270,000 compounds. The 160 analogues were tested for inhibition of ROR1 activity and 19 of them were found to be active. The 160 analogues were also subjected to analytical assays (identity, purity, chemical stability and kinetic solubility using reversed phase HPLC with UV and ESI-MS detection).

Structure-Activity Relationship (SAR) was analyzed for the prioritized clusters and singletons. Synthetic routes were worked out including availability of starting materials. SciFinder novelty searches were carried to analyze the intellectual property (IP) position around the clusters and singletons.

Two singletons, KAN0173631 and KAN0242909, seemed to be interesting for a chemistry program (Supplementary information II, Table 1). The program was focused on KAN0173631 which showed the most promising characteristics with regard to both ability to induce apoptosis of CLL cells and selectivity towards PBMC from healthy donors.

KAN0173631 is a small compound (MW=245 Da) with opportunities of modification at several positions. More than 2000 analogues to KAN0173631 have been synthesized and tested for activity in cell viability assays (PBMCs from CLL patients and healthy volunteers). The cell viability assay has been SAR-driving assay. Selected compounds have been further characterized for effects on ROR1 phosphorylation, apoptosis (Annexin V/PI) as well as for downstream apoptotic signalling molecules. The chemistry program and selection process resulted in a promising lead compound, KAN0439834, with a molecular weight of 530 Da. KAN0439834 has been shown to selectively kill CLL cells, but not normal PBMC, dephosphorylate ROR1 and downstream signaling molecules of the non-canonical Wnt pathway considered to be associated with ROR1 signaling.

A schematic presentation of the development and production of small molecule ROR1 inhibitors is shown in Supplementary information II, Figure 1.

**Supplementary Methods and Information II, Table 1.** Summary of prioritized clusters and singletons following HTS, hit confirmation and analogue testing. LE = ligand efficiency (LE=pIC50/MW), IP = intellectual property, SAR=Structure-Activity Relationship. Cell activity was estimated on representative active compounds from each cluster using Annexin-V/PI on PBMCs from 5 CLL patients and from 5 healthy volunteers.

| **Cluster  number** | **Number of primary HTS hits in cluster** | **Number of tested analogues** | **Number of cpds with  IC_50_ value** | **IC_50_ range (µM)** | **Average Hill slope** | **Average MW (cpds with IC_50_ value)** | **Comments** |
| --- | --- | --- | --- | --- | --- | --- | --- |
| 5 | 3 | 10 | 5 | 1.9 - 9.6 | 1.05 | 288 | High LE and solubility. Straightforward chemistry. Very difficult IP situation. Emerging SAR. Low cell activity. |
| 11 | 2 | 12 | 3 | 4.7 - 20 | 0.91 | 279 | Low solubility. Difficult chemistry. Emerging SAR. No cell activity. |
| 13 | 2 | 11 | 3 | 13 - 47 | 1.32 | 256 | Low potency. Low solubility. Emerging SAR. No cell activity. |
| 14 | 8 | 46 | 13 | 7.2 - 43 | 1.04 | 436 | Low LE. Straightforward chemistry. Emerging SAR. No cell activity. |
| 26 | 7 | 11 | 9 | 2.0 - 20 | 1.15 | 289 | High ligand efficiency. Difficult IP situation. Straightforward chemistry. Low cell activity. |
| 29 | 4 | 17 | 7 | 2.6 - 51 | 1.15 | 321 | Very difficult IP situation. Straightforward chemistry. Strange SAR. Low cell activity. |
| **Singletons** |  |  |  |  |  |  |  |
| KAN0347675 | 1 | 7 | 3 | 0.11 - 59 | 1.11 | 309 | Very difficult IP situation. Straightforward chemistry. Redox-active. High cell activity but no selectivity against healthy PBMCs. |
| KAN0242909 | 1 | 26 | 1 | 5.8 | 0.89 | 376 | Low solubility. Straightforward chemistry. Not ATP-competitive. Modest cell activity and some selectivity against healthy PBMCs. |
| KAN0173631 | 1 | 17 | 1 | 4.6 | 0.98 | 245 | High LE. Straightforward chemistry. Emerging SAR. Good cell activity and selectivity against healthy PBMCs. |
| KAN0166424 | 1 | 1 | 1 | 13 | 1.16 | 203 | High LE and solubility. Very difficult IP situation. Low cell activity. |

**References:**

1. Lor LA, Schneck J, McNulty DE, Diaz E, Brandt M, Thrall SH, et al. A simple assay for detection of small-molecule redox activity. J Biomol Screen 2007 Sep; 12(6): 881-890.

2. Baell JB, Holloway GA. New substructure filters for removal of pan assay interference compounds (PAINS) from screening libraries and for their exclusion in bioassays. J Med Chem 2010 Apr 8; 53(7): 2719-2740.

3. Copeland R (ed). Evaluation of Enzyme Inhibitors in Drug Discovery. Wiley, 2013.

4. Shoichet BK. Screening in a spirit haunted world. Drug Discov Today 2006 Jul; 11(13-14): 607-615.

**Supplementary Methods and Information II, Figure 1. S**chematic presentation of the development and production of small molecule ROR1 inhibitors.

**Supplementary Figures**

**Supplementary Figure 1A.** Apoptosis (%) (Annexin V/PI) of CD45^+^ ROR1^+^ CLL cells before and after incubation with KAN0439834.

**Supplementary Figure 1B.** Apoptosis (%) (Annexin V/PI) induced by KAN0439834
(250 nM) (24h) in T and B cells from a CLL patient and healthy control.

**Supplementary Figure 1C.** Apoptosis (%) (mean±SEM) (Annexin V/PI) induced by KAN0439834 (250 nM) compared to venetoclax (50 nM) using purified B- and T-cells from healthy donors (n=10) incubated *in vitro* for 24 h.

**
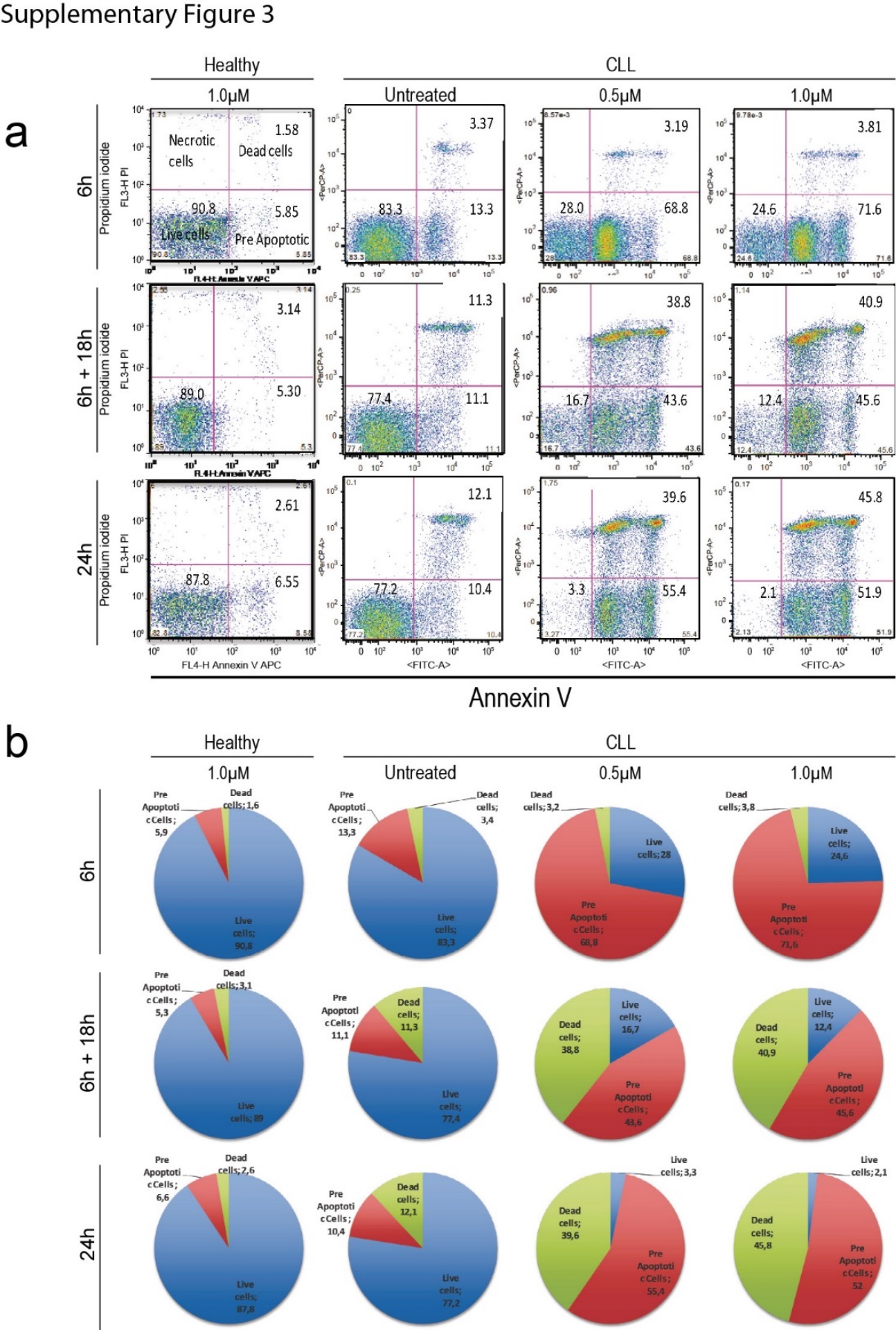
**

**Supplementary Figure 2.** Apoptosis (%) (Annexin V/PI) in CLL cells and PBMC of healthy donors treated with KAN0439834 for various time periods. (**a**) Representative flow cytometry graph of CLL cells and healthy PBMC treated with KAN0439834 for 6 h followed by a wash and then 18 h without drug or incubated for 24 h continuously with KAN0439834. (**b**) The same results shown in Pai diagrams: live cells (blue), pre-apoptotic cells (red) and dead cells (green).

|  | A |
| --- | --- |
|  | B |

**Supplementary Figures 3A and B.** Treatment of CLL cells with Wnt5a and KAN0439834 alone and in combination. (**A**) CLL cells were incubated with or without Wnt5a (25 and 50 ng/ml) and/or KAN0439834 (50 and 250 nM) for 1 h. Western blots show phosphorylation level of ROR1 and SRC before and after incubation with KAN0439834 and recombinant Wnt5a. (**B**) Relative intensity of pROR1/ROR1 (■) and pSRC/SRC (□) following incubation of CLL cells.

**
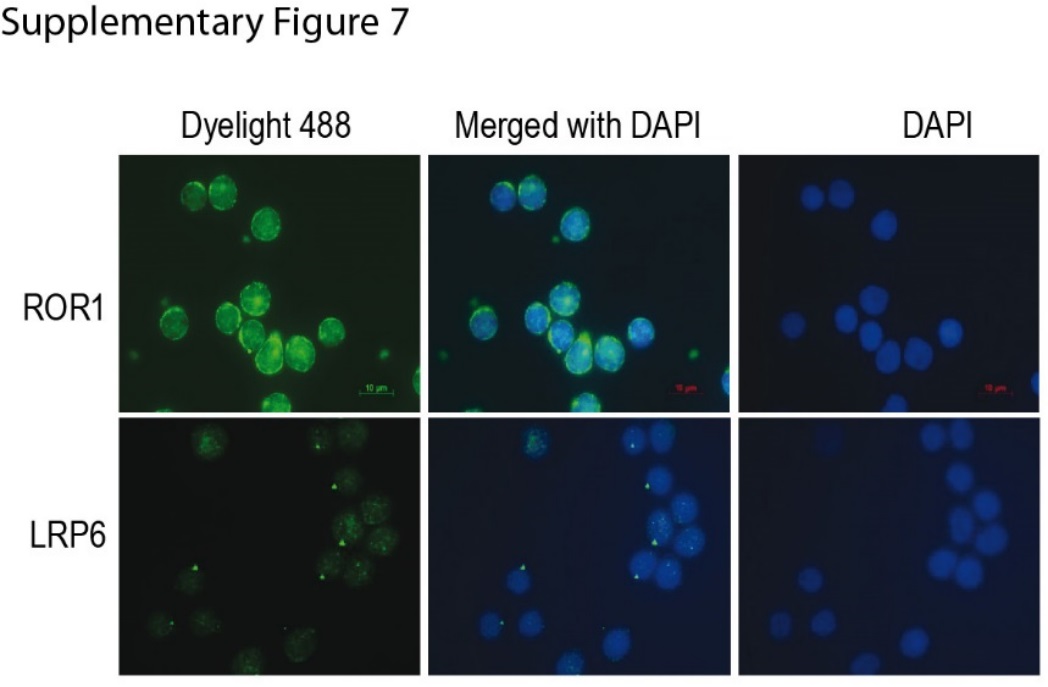
**

**Supplementary Figure 4A.** Immunofluorescent staining. Staining of fresh untreated CLL cells using anti-ROR1 and anti-LRP6 antibodies (63X) (Scale bar: 10 µm).

**
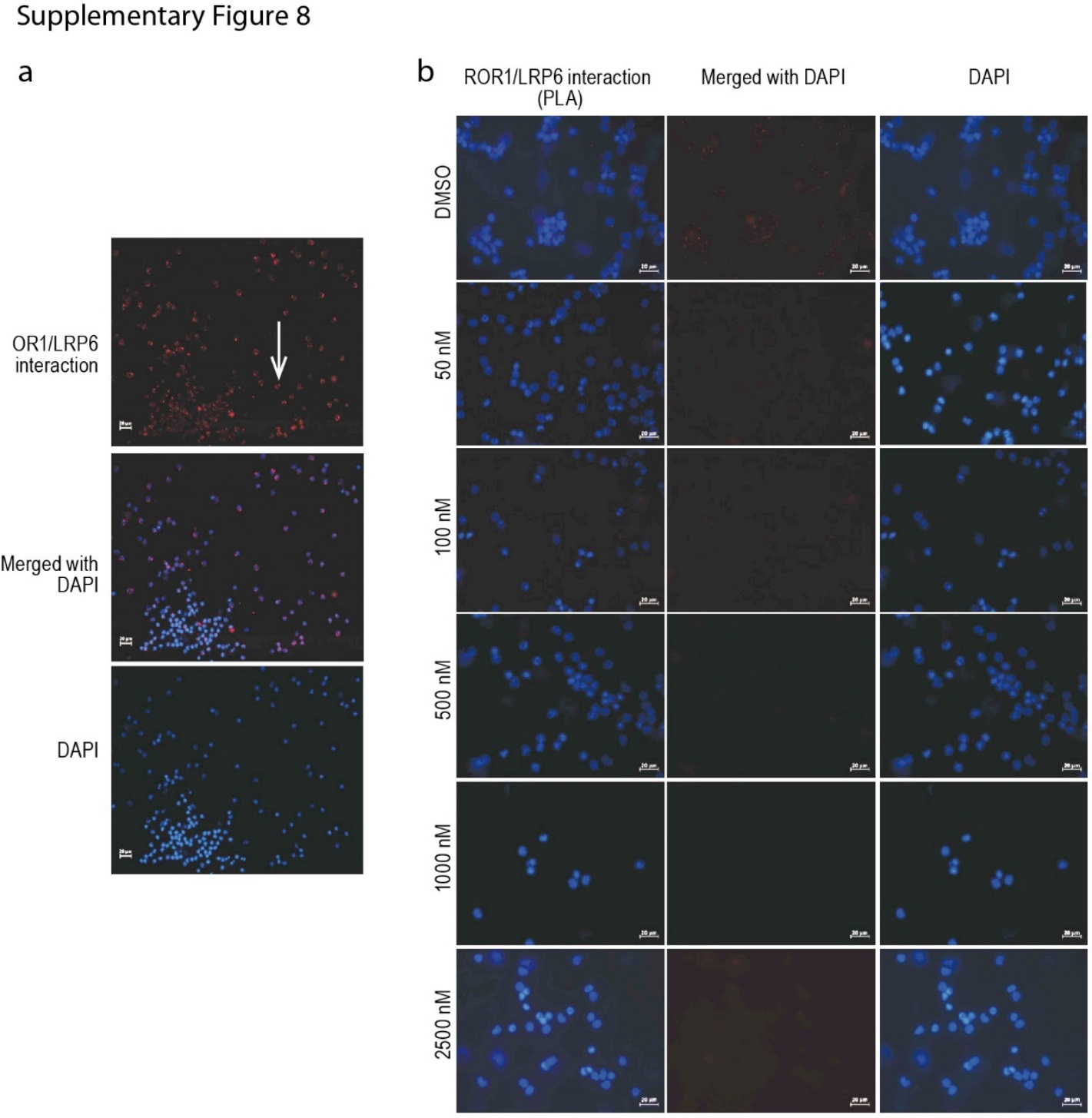
**

**Supplementary Figure 4B.** ROR1/LRP6 dimerization of CLL cells. (**a**) Representative staining images of fresh untreated CLL cells using anti-ROR1 and LRP6 antibodies in the *in situ* proximity ligation assay (PLA) (20X). (**b**) Staining of fresh CLL cells treated with KAN0439834 (50-2500 nM, 6 h) using anti-ROR1 and LRP6 antibodies using *in situ* PLA. DMSO was used as control. Blue fluorescence (DAPI) was used for counterstaining. Pictures were captured by a fluorescent microscope (40X) (Scale bar: 20 µm). Arrows indicate red signals that each represents one ROR1/LRP6 heterodimer.


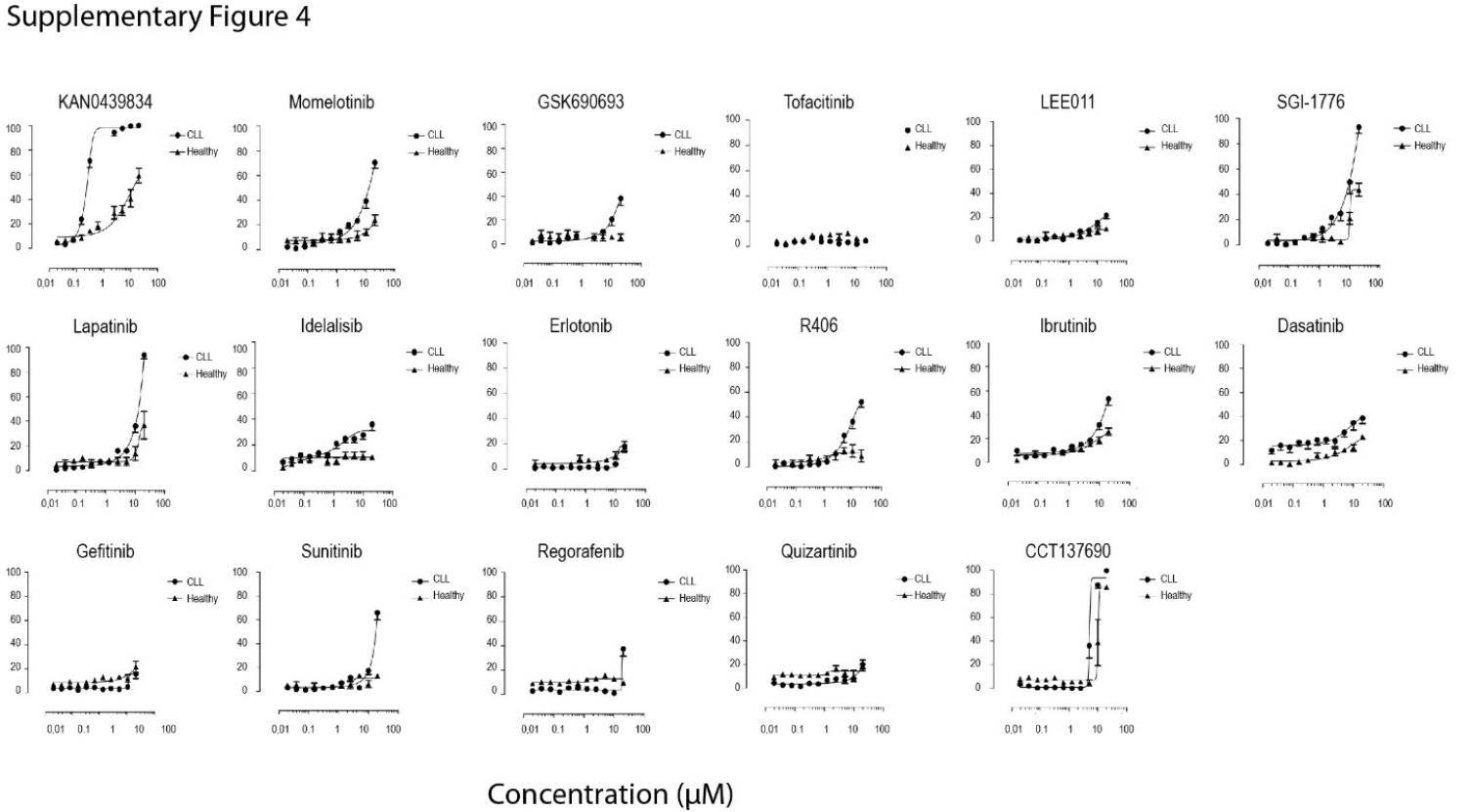


**Supplementary Figure 5.** Cytotoxicity (%) (mean±SEM) (Cell Titer Blue assay) of KAN0439834 in CLL cells compared to non-ROR1 TKIs. Target for each drug is written within brackets: Momelotonib (JAK), GSK690693 (AKT), tofacitinib (JAK), LEE011 (CDK4/6), SG1-1776 (PIM), lapatinib (EGFR and HER2), idelalisib (PI3K), erlotinib (EGFR), R406 (Syk), ibrutinib (BTK), dasatinib (Bcr-Abl), gefitinib (EGFR), sunitinib (multi-RTK), regorafenib (VEGFR2), quizartinib (FLT3), and CT137690 (Aurora). CLL samples (n=8) and healthy PBMC (n=4).


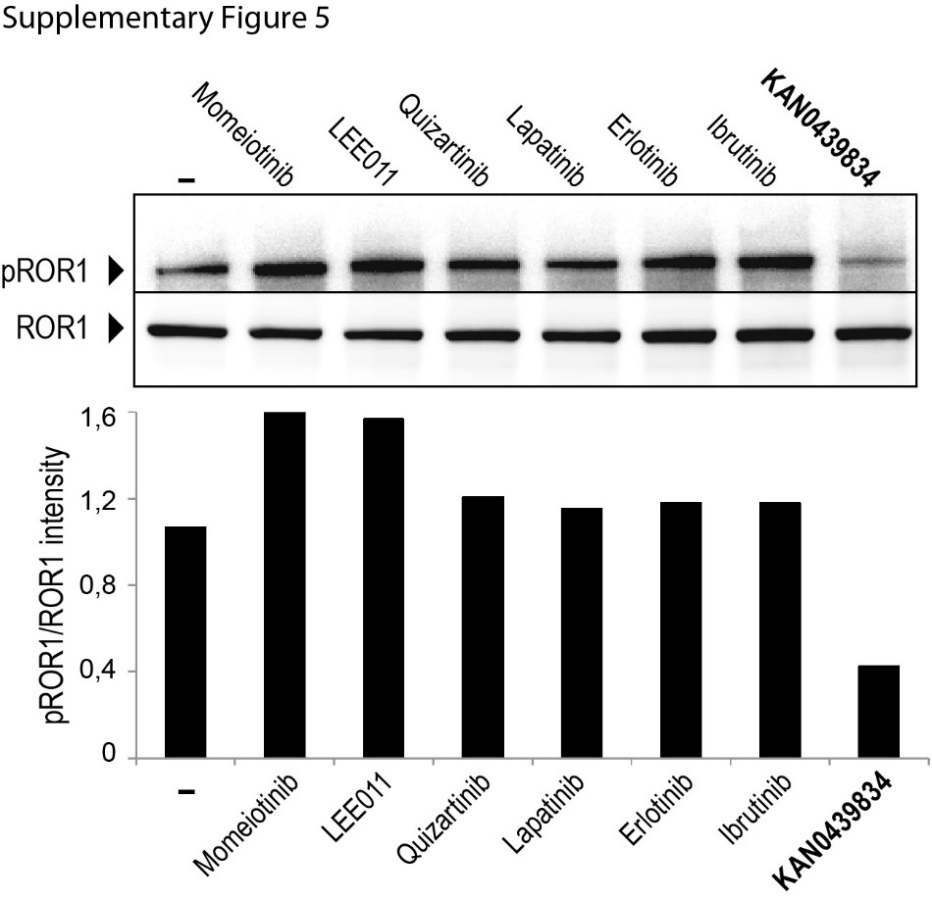


**Supplementary Figure 6A.** ROR1 dephosphorylation (30 min incubation of CLL cells) induced by KAN0439834 (250 nM) or by non-ROR1 TKIs, including momelotinib, LEE011, quizartinib, lapatinib, erlotinib, and ibrutinib (250 nM). Histogram plots show relative intensity of pROR1/ROR1 after incubation.


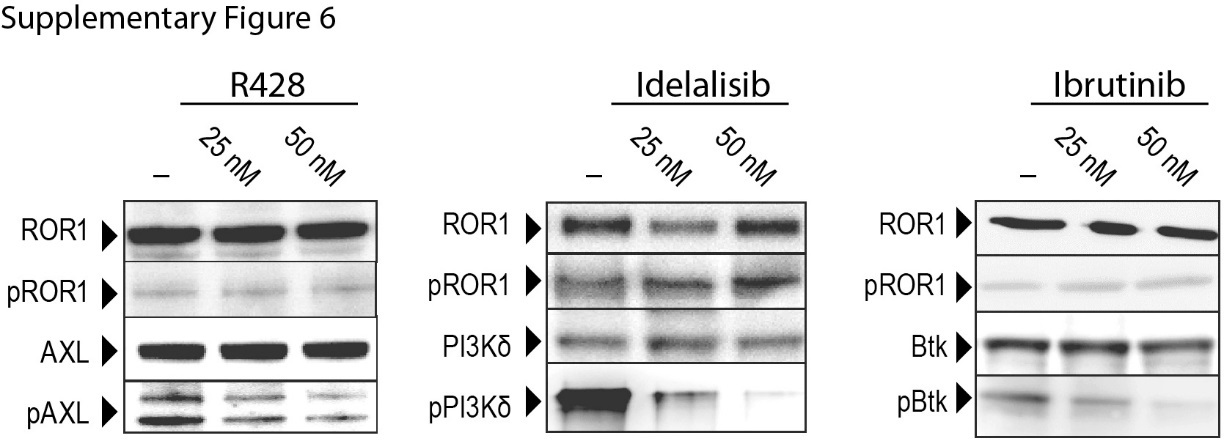


**Supplementary Figure 6B.** Dephosphorylation of ROR1 by R428 (AXL inhibitor), idelalisib (PI3Kδ inhibitor) and ibrutinib (BTK inhibitor) and their respective target after 1 h incubation of CLL cells *in vitro*.


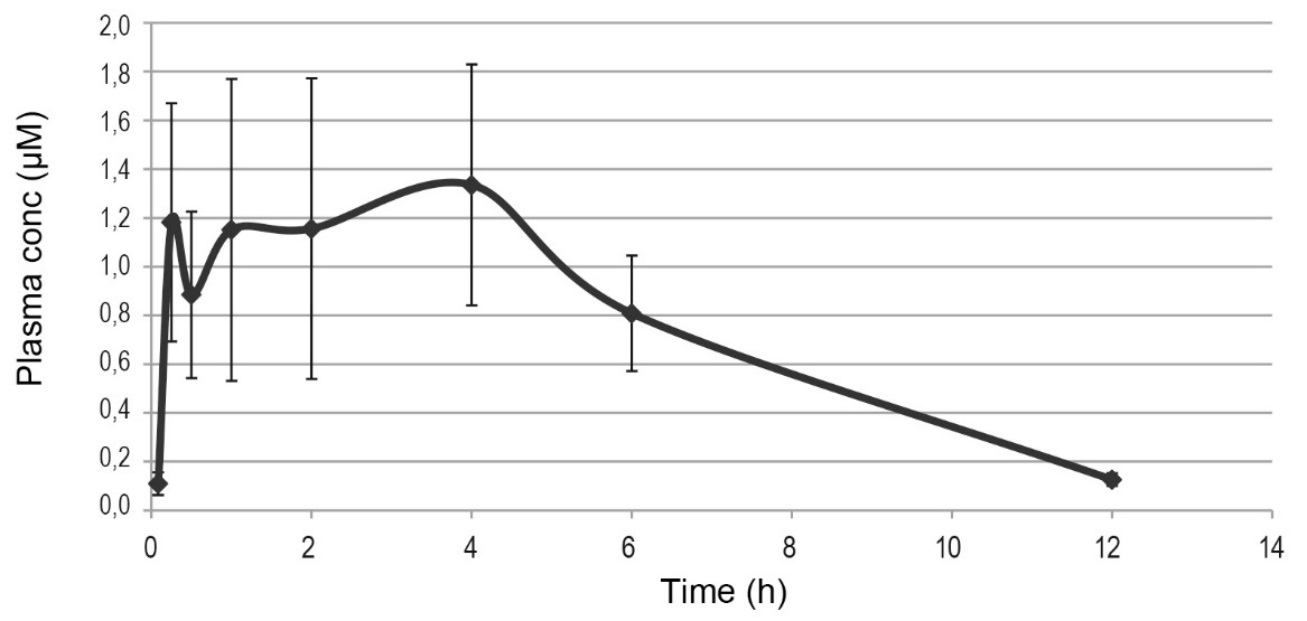


**Supplementary Figure 7.** Pharmacokinetics in CD1 mice after a single oral dose of KAN0439834 (50 mg/kg suspension). 3 samples/time points (in total 9 mice). Plasma concentrations were determined by LC-MS/MS.

.


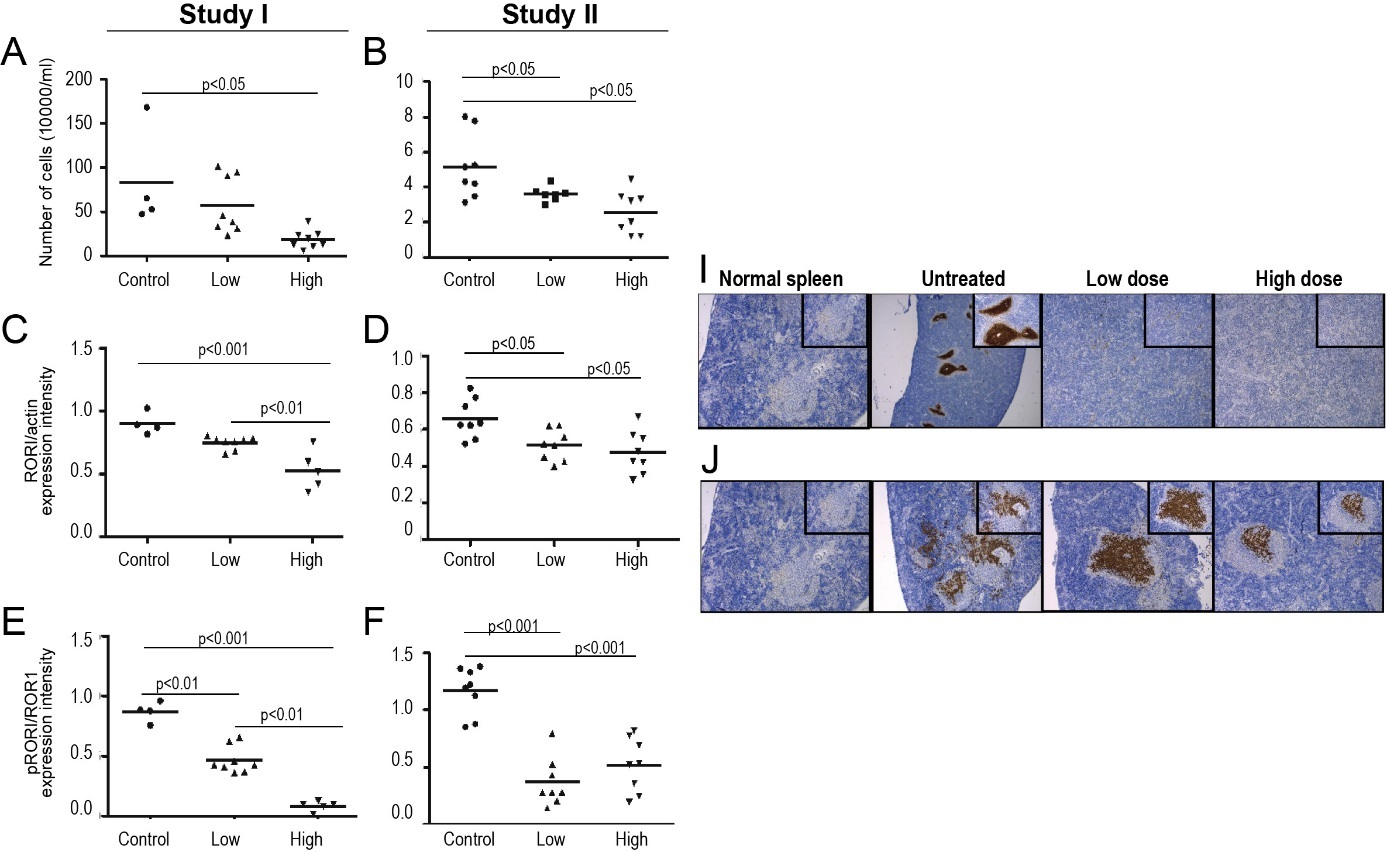


**Supplementary Figure 8.** Effects of oral treatment with KAN0439834 in NOD-SCID mice transplanted with human CLL cells. In study I, CLL cells from a patient with progressive disease without 17p deletion (no p53 mutations) were transplanted into NOD-SCID mice and in Study II CLL cells from a patient with progressive disease with 17p deletion. Treatment started 7 days after transplantation and was continued for 7 days in study I and 14 days in study II. The control groups received only the vehicle. In study I a solution of KAN0439834 was administered orally at a total dose of 10+10 mg/kg daily (low dose) or 25+12.5 mg/kg daily (high dose) for 7 days. In study II the mice received a suspension of KAN439834 orally at a total of 60 mg/kg daily during 14 days (low dose) or 40+40 mg/ml daily during 7 days followed by 60 mg/ml daily for the 7 remaining days. After 7 (study I) or 14 (study II) days, blood was collected and the mice were sacrificed and tissues separated. Half of each spleen was used for preparation of cell suspensions analysed by flow cytometry and the other half were paraffin-embedded (FFPE) and used for immunohistochemistry (IHC). (**a and** **b**) CD45^+^/CD19^+^/ROR1^+^ CLL cells. (**c and d**) ROR1 expression. (**e and f**) phosphorylated ROR1. Significant levels are shown at the top of the graphs. IHC slides (**i and j**) at low (10x) and high (20x) (upper right) resolution. Brown cells represent staining with the human anti-CD45 antibody. Normal mice spleens were negative for the anti-human CD45 antibody
